# Draft Genome Sequence of an Alphaproteobacterium Associated with the Mediterranean Sponge *Oscarella lobularis*

**DOI:** 10.1128/genomeA.00977-15

**Published:** 2015-09-03

**Authors:** Cyril Jourda, Sébastien Santini, Caroline Rocher, André Le Bivic, Jean-Michel Claverie

**Affiliations:** a Aix-Marseille Université, CNRS UMR 7256 (IMM FR 3479), Structural and Genomic Information Laboratory, Marseille, France; b Aix-Marseille Université, CNRS UMR 7263, Mediterranean Institute of Marine and Continental Biodiversity and Ecology (IMBE), Station Marine d’Endoume, Marseille, France; c Aix-Marseille Université, CNRS UMR 7288, Developmental Biology Institute of Marseille Luminy (IBDM), Marseille, France; d Assistance Publique des Hopitaux de Marseille, Marseille, France

## Abstract

While sequencing DNA purified from the homoscleromorph sponge *Oscarella lobularis*, we detected a large number of reads with strong similarity to available alphaproteobacteria gene sequences of family *Rhodobacteraceae*. Here, we present the genome sequence of this putative sponge symbiont that we propose to designate as “*Candidatus* Rhodobacter lobularis.”

## GENOME ANNOUNCEMENT

Sponges (*Porifera* phylum) are metazoans divided into four major classes, including *Homoscleromorpha*, *Demospongiae*, *Hexactinellida*, and *Calcarea*. *Oscarella lobularis* is a *Homoscleromorpha* sponge endemic to the Mediterranean Sea which has several color morphs and host four morphotypes of microbes with dominance of the *Alphaproteobacteria* species (1). Here, we present the draft genome sequence of a new member of the *Rhodobacteraceae* family found in high abundance associated to *Oscarella lobularis* sampled in cave Endoume, Marseille, France.

We initiated the genome sequencing using Illumina technology with DNA-seq paired ends and Nextera mate-pair protocols on a HiSeq2500 sequencer. Low-quality read ends (*Q* < 28), adapter, and cloning vector sequences were trimmed and short remaining sequences (< 100 pb) were removed using Cutadapt (2). The remaining 112 million paired reads were assembled with IDBA-UD (3) and the scaffolding tool from the Platanus assembler (4). Ends and gaps within scaffolds were tentatively closed using GapFiller (5). Extended scaffolds were assembled using CAP3 (6). Read pairs were mapped onto scaffolds using Bowtie (7) and sequencing coverage levels were estimated for each scaffold using SAMtools (8). All 16S sequences were retrieved and taxonomically annotated following homology searches run on the SILVA database (9).

A typical 16S *Rhodobacteraceae*-like sequence was determined at a high coverage rate (1,005×), approximately corresponding to 20 bacterial cells per sponge cell. We propose “*Candidatus* Rhodobacter lobularis” as a name for this new species based on its 16S taxonomic classification and its association with the sponge. The two large scaffolds corresponding to this species were pointed out by their high sequencing coverage (>1,070× in average) and further characterized through a similarity search of their predicted genes (using MetaGeneMark [10]) on the NR database (best BLASTp hits, *E* < 10^−5^). The resulting 5,034,992-bp draft genome sequence is G+C rich (63.5%). The genome assembly was annotated with the Rapid Annotation using Subsystems Technology (RAST) server (11). Annotations of protein encoding genes were validated using homology searches on GenBank, eventually improved using Artemis (12). A total of 47 tRNA genes and 4,787 proteins were predicted, corresponding to 85.75% of the assembled sequence. We identified all *Alphaproteobacteria* core genes (13), suggesting that the present sequence is nearly complete.

Of the predicted proteins, 3,523 (73%) were assigned to gene families using OrthoMCL algorithm and database (14) and 2,416 (50.5%) were assigned to biological KEGG pathways using the KAAS server (15). As expected, “*Ca.* Rhodobacter lobularis” harbors the core metabolic pathways such as glycolysis, gluconeogenesis, the tricarboxylic acid cycle (TCA), and the pentose phosphate pathway. The genome also exhibits a tandem cluster of gene transfer agents, the bacteriophage-like elements that mediate horizontal gene transfer found in nearly all *Rhodobacterales* (16).

Comparison with genome sequences available in RAST showed that *Phaeobacter gallaeciensis* (score, 518), *Sagittula stellata* (score, 509), *Roseobacter* sp*.* AzwK-3b (score, 503), *Rhodobacterales bacterium* Y4I (score, 489), *Roseobacter* sp. SK209-2-6 (score, 482), *Silicibacter pomeroyi* (score, 452), and *Ruegeria pomeroyi* DSS-3 (score, 438), all *Rhodobacterales*, were the closest neighbors of this new species.

### Nucleotide sequence accession numbers.

The whole-genome shotgun project has been deposited at DDBJ/EMBL/GenBank under the accession no. LFTY00000000. The version described in this paper is the first version, LFTY01000000, and consists of the two sequences LFTY01000001 and LFTY01000002.
